# Development and validation of an explainable machine learning model for differentiating diabetic nephropathy from diabetic retinopathy in patients with type 2 diabetes

**DOI:** 10.3389/fendo.2026.1902508

**Published:** 2026-09-03

**Authors:** Yonglin Zhang, Siyu Feng, Yukun Xue, Li Xue, Jiesi Luo

**Affiliations:** 1 Department of Pharmacy, Affiliated Hospital of North Sichuan Medical College, Nanchong, Sichuan, China; 2 Department of Public Health, Nanchong Mental Health Center Of Sichuan Province, Nanchong, Sichuan, China; 3 School of Public Health, Southwest Medical University, Luzhou, Sichuan, China; 4 School of Basic Medical Science, Southwest Medical University, Luzhou, Sichuan, China

**Keywords:** diabetic complications, machine learning, prediction model, SHAP, type 2 diabetes mellitus

## Abstract

**Background:**

Diabetic nephropathy (DN) and diabetic retinopathy (DR) are common microvascular complications of type 2 diabetes mellitus (T2DM) and may require different diagnostic and management pathways. This study aimed to develop and validate an interpretable machine learning model based on routine laboratory data to differentiate prevalent DN from prevalent DR among hospitalized patients with type 2 diabetes.

**Methods:**

Data were collected from a large tertiary hospital in China and split into a training/internal validation cohort (DN: 2,309 cases; DR: 855 cases) and an independent held-out validation cohort (DN: 578 cases; DR: 214 cases). A total of 47 routinely available laboratory and demographic variables were extracted from electronic health records (EHRs). Seven machine learning algorithms were developed and compared, with recursive feature elimination (RFE) employed to identify the most informative subset of features and enhance model performance and interpretability. Model discrimination was assessed using the area under the receiver operating characteristic curve (AUC) and the area under the precision-recall curve (AP), while SHAP values were used to interpret feature importance and explain individual-level predictions.

**Results:**

The extreme gradient boosting (XGBoost) classifier demonstrated the highest predictive performance among the seven machine learning algorithms evaluated. After selecting the top five features based on importance rankings, an explainable XGBoost model was constructed. This final model achieved strong apparent discrimination in both the training/internal validation cohort (AUC = 0.991, 95% CI: 0.989-0.994; AP = 0.979, 95% CI: 0.973-0.984) and the held-out validation cohort (AUC = 0.997, 95% CI: 0.996-0.999; AP = 0.993, 95% CI: 0.988-0.997). SHAP analysis further identified α-hydroxybutyrate dehydrogenase, creatine kinase-MB, creatinine, urinary α1-microglobulin, and N-acetyl-β-D-glucosaminidase as the most influential features contributing to complication risk prediction.

**Conclusions:**

An explainable machine learning model for predicting complications in patients with T2DM demonstrated high feasibility and effectiveness, indicating strong potential to support clinical management and improve patient outcomes. By incorporating SHAP analyses, the model addresses key concerns regarding transparency and clinical decision-making. These findings highlight the model’s potential for real-world clinical implementation.

## 1 Introduction

The T2DM is one of the most widespread and burdensome chronic diseases worldwide (1–3). According to the International Diabetes Federation, the number of individuals affected has surpassed 530 million and is projected to reach 780 million by 2045 (4). The disease imposes a particularly heavy burden in developing countries such as China, which now has the largest diabetic population globally, with rising prevalence observed even among younger adults (5). While hyperglycemia itself poses metabolic risks, the long-term clinical and societal impact of T2DM arises primarily from its chronic complications, which account for the majority of morbidity, mortality, and healthcare costs associated with the disease (6–8).

Among the various complications of T2DM, microvascular diseases such as DN and DR are particularly prevalent and clinically significant. DN is a major contributor to the development of end-stage renal disease (9, 10), while DR remains one of the leading causes of preventable blindness in working-age adults (11–13). These two conditions not only represent key endpoints in diabetes-related morbidity but are also associated with considerable economic burden and reduced quality of life. Importantly, DN and DR often share overlapping risk factors, including hyperglycemia, hypertension, dyslipidemia, and long disease duration, and may occur concurrently in the same patient. Evidence from epidemiological and clinical studies suggests a strong correlation between the presence of DR and the future development of DN, indicating potential value in combined risk assessment strategies (14–17). Despite these links, DN and DR differ markedly in their clinical manifestations, progression rates, monitoring requirements, and treatment strategies (18–20). For instance, DR requires timely ophthalmologic evaluation and potential intravitreal therapy, whereas DN necessitates renal function monitoring, blood pressure control, and nephroprotective intervention.

As such, early differentiation between these complications is essential to ensure appropriate and timely care tailored to the specific disease process (21–24). However, conventional diagnostic tools remain limited, renal biopsy is invasive and unsuitable for routine screening (25, 26), while fundus examinations depend heavily on operator expertise and may miss subtle or early retinal changes (27). Given these challenges, there is growing interest in leveraging routinely collected clinical data to support early, non-invasive risk stratification of diabetic complications (28–30). Identifying patients at high risk for DN or DR using objective, data-driven methods could enable more proactive management, improve long-term outcomes, and optimize the allocation of healthcare resources.

The objective of this study was to develop and validate an interpretable machine learning model using routinely collected laboratory data to differentiate between hospitalized T2DM patients with prevalent DN and DR. The model is intended to support differential diagnosis and clinical triage for patients with established or suspected diabetic microvascular complications. To achieve this, various machine learning algorithms were constructed and evaluated on a retrospective cohort, optimizing feature combinations to enhance performance. Model stability and diagnostic accuracy were further evaluated in the independent held-out validation cohort, and the best-performing model was selected as the final model. Its diagnostic accuracy in distinguishing DN from DR was assessed, demonstrating potential clinical utility. To ensure transparency, feature contributions were interpreted using SHAP values. Ultimately, the development of this model may optimize clinical decision-making and enable more timely, individualized management of diabetic microvascular complications in hospitalized T2DM patients.

## 2 Methods

### 2.1 Study design and participants

This risk model development and validation study was conducted using EHR data from the Affiliated Hospital of North Sichuan Medical College. The EHR data contains comprehensive clinical information, including laboratory test results, diagnostic codes, medication prescriptions, and physician notes. All laboratory data used in the analysis were obtained from ISO 15189-accredited laboratories. Ethical approval for the study was obtained from the Medical Ethics Committee of the Affiliated Hospital of North Sichuan Medical College (Approval No. 2023ER336-1). Given the retrospective design of the study and the fully de-identified nature of the dataset, the requirement for written informed consent was formally waived by the committee. This study was reported in accordance with the TRIPOD (Transparent Reporting of a multivariable prediction model for Individual Prognosis Or Diagnosis) guidelines (**Supplementary Table 1**) (31).

A total of 128 routine clinical parameters were collected at the time of initial disease evaluation, corresponding to the first available blood and urine test results recorded by the hospital laboratory. To minimize treatment-related bias, patients who had received any disease-modifying therapy or other medical interventions prior to blood or urine sampling were excluded. Parameters with more than 25% missing values were excluded from further analysis. For parameters with less than 25% missing data, values were imputed using the Multiple Imputation by Chained Equations (MICE) method. Feature selection was subsequently performed using the Least Absolute Shrinkage and Selection Operator (LASSO) logistic regression model to identify statistically significant variables. To reduce redundancy, pairwise Spearman correlation coefficients were calculated among the selected parameters, and hierarchical clustering was applied. Within each resulting cluster, the parameter with the highest average correlation to other members was selected as the representative variable. Following this multistep data preprocessing procedure, 47 clinical parameters were retained for model development. These included 6 hematological parameters, 13 biochemical parameters, 4 coagulation-related parameters, 6 inflammatory or immunological parameters, 5 urine-based parameters, 8 enzyme- or metabolism-related parameters, and 5 classified as other. These selected parameters were subsequently used to construct predictive models based on a labeled dataset of patients with diabetic nephropathy or retinopathy.

To minimize data leakage, data partitioning was performed at the patient level before model development. The final dataset consisted of 3,956 patients with type 2 diabetes, including 2,887 with DN and 1,069 with DR. Patients diagnosed with both DN and DR were excluded to ensure that the analysis focused on distinguishing the two complications independently and to reduce potential confounding from overlapping phenotypes. The dataset was divided into two mutually exclusive cohorts for model development and independent evaluation. Specifically, 80% of the data (2,309 DN cases and 855 DR cases) were assigned to the training/internal validation cohort for model training and hyperparameter tuning, whereas the remaining 20% (578 DN cases and 214 DR cases) were reserved as an independent held-out validation cohort. This held-out validation cohort was an internal subset derived from the same source population and was used exclusively for final evaluation of model performance on unseen data. Feature selection using RFE was conducted only within the model-development cohort. The held-out validation cohort was not used for feature selection, hyperparameter tuning, model training, preprocessing parameter estimation, or threshold optimization. All preprocessing procedures were fitted on the training data and then applied unchanged to the corresponding non-training data.

### 2.2 Model benchmarking

Seven predictive models based on conventional machine learning algorithms were developed, including adaptive boosting (AdaBoost), decision tree (DT), k-nearest neighbor (KNN), logistic regression (LR), random forest (RF), support vector machine (SVM), and XGBoost. All models were implemented using the XGBoost Python package (version 2.1.1) and scikit-learn (version 1.5.2).

To minimize optimistic bias in internal validation, model training and hyperparameter tuning were performed using a nested cross-validation strategy within the training cohort. Specifically, an outer five-fold stratified cross-validation was used to estimate predictive performance, while Bayesian hyperparameter optimization was conducted within each outer training fold using an inner five-fold stratified cross-validation, implemented with the scikit-optimize library (version 0.10.2). The search space for each model was defined based on common best practices in clinical prediction modeling. For tree-based models (XGBoost, AdaBoost, RF, and DT), hyperparameters such as the number of estimators (ranging from 50 to 500), maximum tree depth (3 to 50), learning rate (0.01 to 0.3), minimum samples for node splitting (2 to 10), and splitting criteria (gini or entropy) were tuned. For LR, regularization strength (0.01 to 100), penalty type (L1 or L2), and solver method (liblinear or saga) were explored. The SVM model was tuned over the regularization parameter *C* (0.01 to 32), kernel function (rbf), and kernel coefficient (gamma or scale). For KNN, the number of neighbors (3 to 50), distance metric (Minkowski with p = 1 to 3), and weighting function (uniform or distance) were optimized. All models were trained using a fixed random seed to ensure reproducibility.

Model performance was evaluated using both the training/internal validation cohort and the independent held-out validation cohort. The primary evaluation metrics included the area under the receiver operating characteristic (ROC) curve (AUC) and the area under the precision-recall (PR) curve, quantified as average precision (AP). A two-stage validation strategy was implemented to ensure robust and unbiased evaluation. In the first stage, training/internal validation were performed through five-fold cross-validation on the training cohort. To account for variability across folds, the mean and 95% confidence interval (CI) of each evaluation metric were calculated from the cross-validation results. In the second stage, performance was assessed on the independent held-out validation cohort, which was strictly excluded from all stages of model training and hyperparameter tuning. For this cohort, 95% CIs for AUC and AP were estimated using 1,000 iterations of non-parametric bootstrap resampling with replacement.

### 2.3 Variable importance and model interpretation

Model interpretability was evaluated using SHapley Additive exPlanations (SHAP, version 0.48.0), which quantify the contribution of each variable to the model output based on a game theoretical Shapley value (32, 33). Higher SHAP values indicate greater influence on the model-predicted risk. SHAP not only reflects the magnitude of a variable’s impact but also its direction, identifying whether it increases or decreases the predicted probability. Mean absolute SHAP values were calculated to provide a global summary of variable importance, while SHAP decision plots were generated to illustrate how individual predictions were influenced by key variables. To further examine the average effect of each variable on model predictions, Accumulated Local Effects (ALE) plots were generated using the scikit-explain package (version 0.1.4). ALE captures local variations in the predicted output across the distribution of each variable, accounting for potential interactions and feature dependencies. This method offers a robust, model-agnostic understanding of nonlinear relationships between variables and outcomes (34).

### 2.4 Model optimization and feature selection

For an optimal subset of variables, RFE was conducted to evaluate how predictive performance varied with different combinations of input variables. Starting with all 47 selected variables, an initial model was trained using five-fold cross-validation, with hyperparameter tuning performed via Bayesian optimization. Variable importance was quantified using a model-specific ranking algorithm tailored to the trained model’s structure. Variables were then ordered by their relative importance, and the least informative variable was iteratively removed. At each step, a new model was retrained using the updated subset of top-ranked variables. This elimination process continued until only a single variable remained. Model performance at each iteration was evaluated using three metrics: accuracy, AUC, and F1-score. The final number of variables retained in the model was determined by identifying the point at which predictive performance remained stable or began to degrade, thereby achieving an optimal balance between model simplicity and classification accuracy.

### 2.5 Final model establishment

The final model was developed using the optimal subset of variables identified through feature selection. Training and validation procedures followed those described in the model benchmarking section. This study was developed and reported in strict adherence to the TRIPOD-AI (Transparent Reporting of a multivariable prediction model for Individual Prognosis Or Diagnosis for Artificial Intelligence) reporting guidelines for machine learning–based clinical prediction model studies (**Supplementary Table 1**). Model performance was assessed on both the training/internal validation cohort and the independent held-out validation cohort using AUC, AP, and their corresponding 95% confidence intervals. A probability threshold of 0.396 was selected to balance classification performance and clinical risk-stratification considerations. Predictions with probability values below 0.396 were classified as DN, whereas those equal to or above 0.396 were classified as DR.

## 3 Results

### 3.1 Study design overview

**Figure 1:**
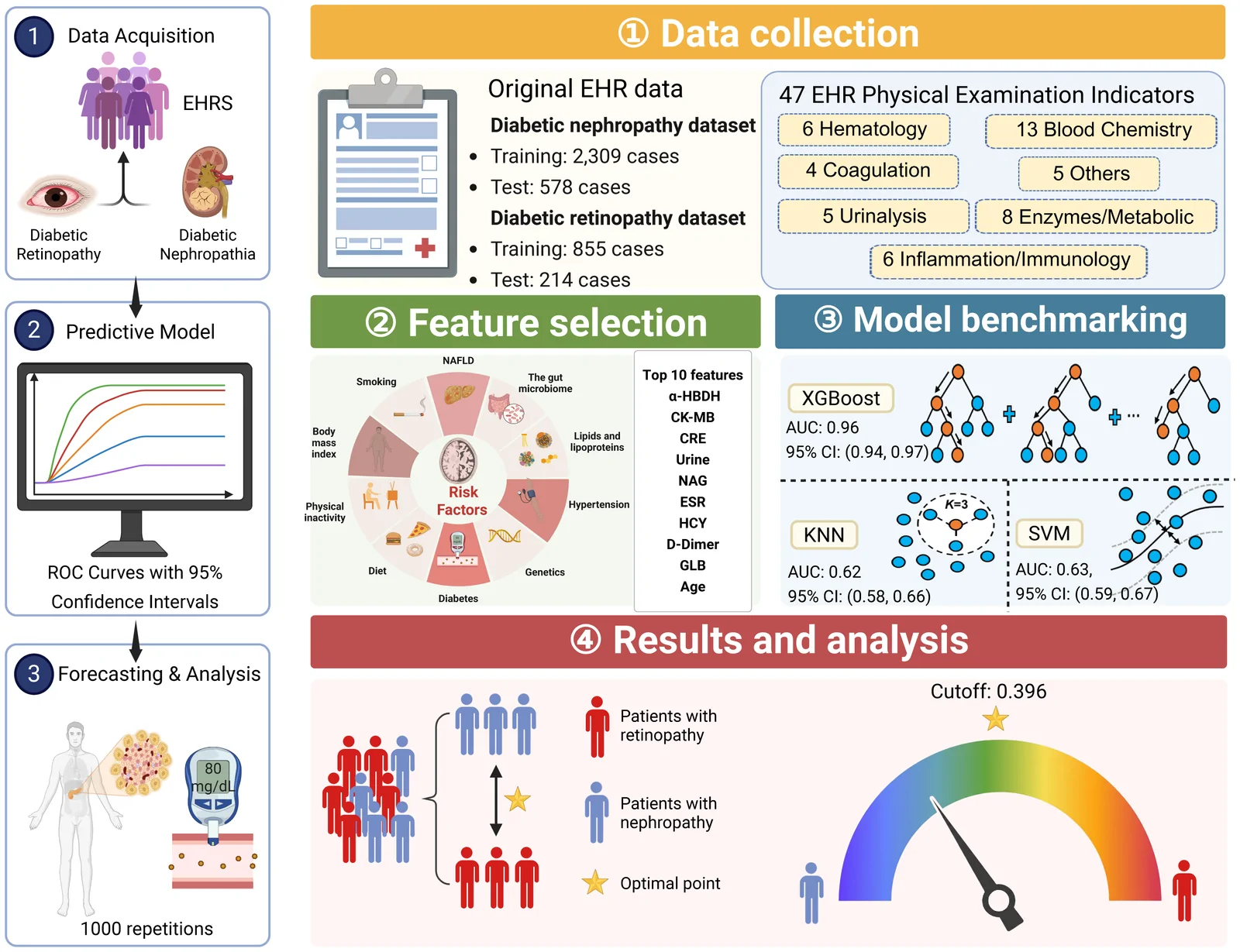
Overall study design for diabetic complication prediction and clinical care guidance.

The overall study framework, including data acquisition and preprocessing, model development and validation, and subsequent interpretability analyses, is summarized in **Figure 1**. Between December 6, 2013, and January 1, 2024, a total of 3,956 patients with type 2 diabetes were enrolled at the Affiliated Hospital of North Sichuan Medical College. All patients were clinically diagnosed with either DN or DR by certified endocrinologists or ophthalmologists according to international diagnostic guidelines. The baseline demographic and clinical characteristics of the DN and DR groups are presented in **Table 1**. The mean baseline age was 62.9 years (standard deviation (s.d.) = 12.1), 46.1% (N = 1,825) were female, and 26.3% (N = 1,039) reported a history of alcohol consumption. Of the total cohort, 3,164 patients (80%) were randomly assigned to the training/internal validation cohort for model development, whereas the remaining 792 patients (20%) were reserved as a held-out validation cohort for independent performance assessment. The training cohort contained 855 events and 2,309 non-events, corresponding to an event rate of 27.0%, while the held-out validation cohort showed a comparable distribution (214 events and 578 non-events; event rate, 27.0%). A broad set of clinical parameters was extracted from EHRs, including hematological, urinary, serum or plasma, and physiological variables. Feature selection was performed to evaluate clinical relevance and to identify a reduced yet informative subset of variables that could facilitate future clinical assay development while preserving predictive performance. In total, 53 candidate predictors were entered into model development, corresponding to an events-per-variable (EPV) of 16.13 and a non-events-per-variable (NPV) of 43.57 in the training cohort, supporting the adequacy of the dataset for model development. Seven machine learning algorithms were then used to develop binary classification models for distinguishing between the two major diabetic complications, including four tree-based ensemble methods (AdaBoost, DT, RF, and XGBoost), two instance-based methods (SVM and kNN), and LR. During model development, class weights were adjusted according to the outcome distribution to reduce potential bias toward the majority class. All models were trained and evaluated using consistent stratified cross-validation folds. Their performance was compared to identify the most suitable classifier for final implementation. The selected model generated probability estimates for each diagnostic outcome, thereby enabling individualized decision-making and supporting subsequent clinical management.

**Table 1:** Comparison of related variables between patients with DN and DR.

Variables	DR	DN	*p*_value
Gender = male	507 (47.4%)	1318 (45.7%)	0.338
Gender = female	562 (52.6%)	1569 (54.3%)	–
Creatine Kinase-MB Activity	11.22 (9.016, 13.706)	10.857 (8.075, 13.706)	0.003
α-Hydroxybutyrate Dehydrogenase	138.138 (114.036, 165.011)	153.348 (121.145, 185.04)	<0.001
Blastomyces Yeast (BYST)	0 (0, 0)	0 (0, 0)	0.029
Pathological Cast (path. CAST)	0 (0, 0)	0 (0, 0)	0.044
Crystal Count (X-TAL)	0 (0, 1)	0 (0, 1)	0.019
Respiratory Rate	20 (20, 21)	20 (20, 21)	0.014
Sodium	141.085 (138.142, 143.31)	140.577 (137.8, 142.8)	0.001
Glycated Hemoglobin A1c	12.3 (9.5, 15.64)	12.07 (9.3, 15.8)	0.675
Potassium	4 (3.692, 4.351)	3.91 (3.568, 4.304)	<0.001
Epithelial Cell Count (EC)	0.88 (0, 3)	1 (0, 3.52)	0.002
Platelet Distribution Width	16.5 (16.2, 16.7)	16.4 (16.1, 16.7)	0.09
Platelets	177 (136, 219)	177 (133, 231)	0.616
Mean Corpuscular Hemoglobin	29.8 (28.7, 30.9)	29.7 (28.5, 30.9)	0.233
Red Cell Distribution Width CV	13.2 (12.8, 13.9)	13.4 (12.8, 14.1)	0.001
Mean Corpuscular Hemoglobin Concentration	328 (319, 334)	326 (318, 333)	0.008
Creatinine	71 (53.9, 98)	93.6 (66.9, 147.8)	<0.001
Homocysteine	10.6 (8.508, 13.1)	11.84 (9.52, 15.007)	<0.001
Monocyte Percentage	5.7 (4.7, 6.9)	5.8 (4.7, 7.2)	0.012
Neutrophil Percentage	67.7 (60.5, 74.7)	69.8 (62.1, 77.9)	<0.001
Eosinophil Percentage	1.6 (0.9, 2.9)	1.6 (0.7, 3)	0.046
Lymphocyte Absolute Count	1.48 (1.12, 1.84)	1.39 (1, 1.81)	<0.001
Urinary α1-Microglobulin	21.354 (8.2, 37.4)	32 (14, 72.2)	<0.001
N-Acetyl-β-D-Glucosaminidase	14.3 (8.8, 20.9)	16.8 (10.5, 26.4)	<0.001
Erythrocyte Sedimentation Rate	19 (7, 42)	28 (11, 54)	<0.001
Plasma Fibrinogen Concentration	3.705 (3.02, 4.749)	4.02 (3.1, 5.185)	<0.001
Albumin	39.4 (34.9, 43.2)	36.855 (32, 40.385)	<0.001
Albumin: Globulin Ratio	1.28 (1.1, 1.49)	1.2 (0.98, 1.42)	<0.001
Hematocrit	0.38 (0.334, 0.418)	0.364 (0.312, 0.409)	<0.001
Plasma D-Dimer	0.8 (0.64, 1.15)	0.97 (0.72, 1.84)	<0.001
Total Calcium	2.29 (2.19, 2.39)	2.25 (2.15, 2.35)	<0.001
Indirect Bilirubin	6.6 (4.7, 8.8)	6.4 (4.3, 8.5)	0.041
Red Blood Cell Count (RBC)	3 (0, 11)	4 (1, 13)	0.003
Urine Creatinine Concentration	5909.7 (4167.1, 9237.7)	5687.6 (4026.15, 8338.95)	0.035
Prothrombin Time	12.1 (11.1, 13)	12.5 (11.5, 13.4)	<0.001
Antithrombin III Activity Assay	102.1 (89.9, 113)	96.8 (85, 107.3)	<0.001
Age	60 (52, 69)	65 (55, 73)	<0.001
High-Density Lipoprotein Cholesterol	1.16 (0.912, 1.47)	1.09 (0.877, 1.38)	<0.001
Low-Density Lipoprotein Cholesterol	2.517 (1.91, 3.27)	2.51 (1.873, 3.25)	0.722
Very Low-Density Lipoprotein Cholesterol	0.7 (0.47, 1)	0.76 (0.53, 1.095)	0.001
Globulins	30.5 (27.4, 34.2)	30.4 (26.8, 35.1)	0.689
Adenosine Deaminase	13.6 (10.68, 18.81)	14.88 (11.44, 19.503)	<0.001
Alkaline Phosphatase	89.65 (70.95, 111.501)	89.856 (70.75, 111.752)	0.433
5’-Nucleotidase	3.4 (2.294, 4.745)	3.5 (2.442, 5.3)	<0.001
Aspartate Aminotransferase	20.6 (16.171, 26.4)	20.9 (16.32, 28.016)	0.033
Complement Clq	192.28 (169.385, 220.79)	194.61 (166.14, 225.04)	0.309
α-L-Fucosidase	18.5 (13.5, 26.1)	18.5 (13.57, 25.69)	0.712

### 3.2 Benchmark of machine learning models for predicting DN and DR

Following the identification of the two diabetic complications and the establishment of training and test cohorts, diverse multi-source patient data were processed and integrated into several machine learning models for performance evaluation. These models were trained to predict the risk of DN and DR using EHR data, which included blood and urine biomarkers, clinical measurements, and medication information relevant to treatment. Missing values for each parameter were estimated and imputed before model training.

**Figure 2:**
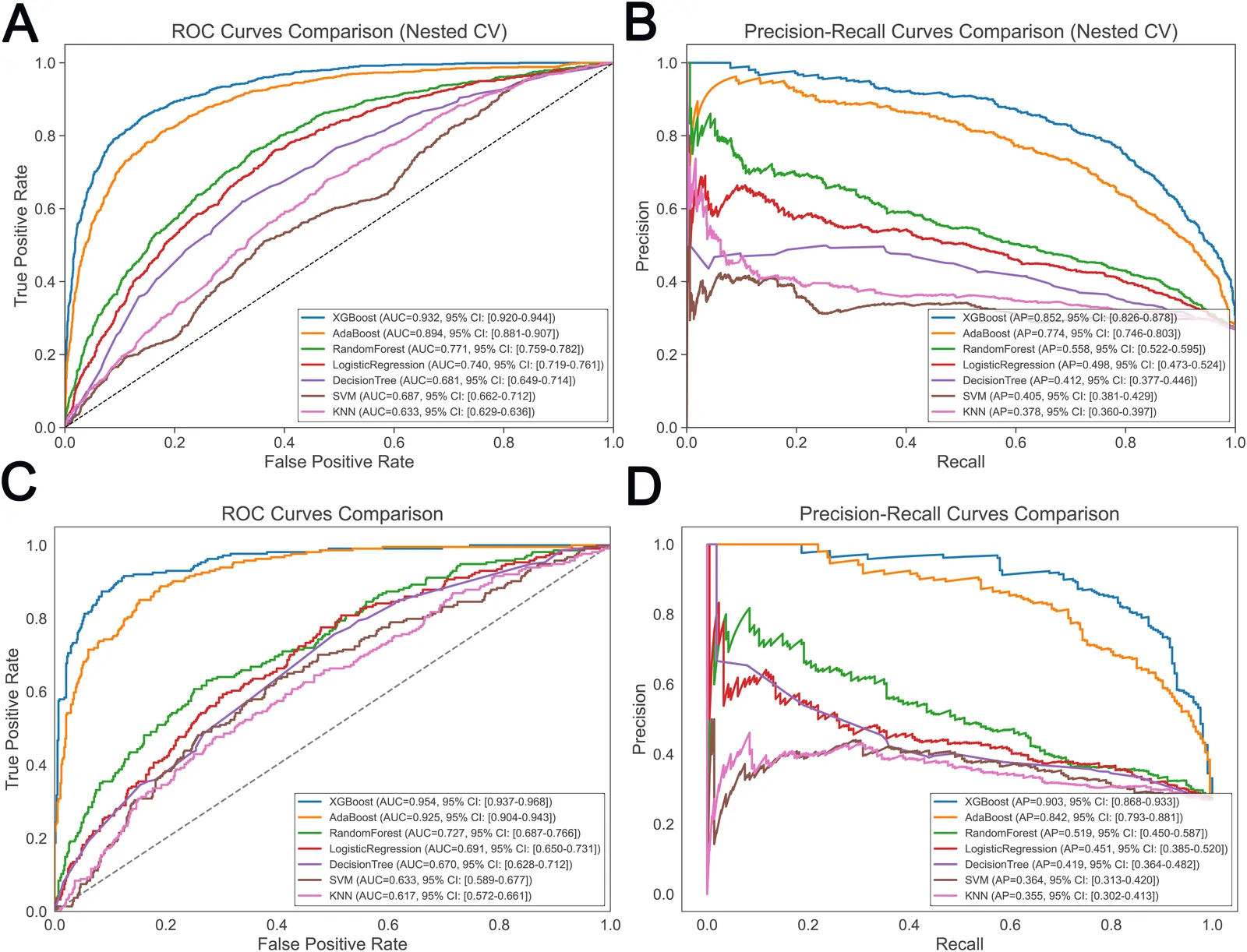
Performance comparison of different models. **(A)** ROC curves and corresponding AUC values, and **(B)** PR curves with corresponding AP values for models evaluated on the training/internal validation cohort. **(C)** ROC curves and corresponding AUC values, and **(D)** PR curves with corresponding AP values for models tested on the independent held-out validation cohort. For the training/internal validation cohort, performance was estimated using nested cross-validation. For the held-out validation cohort, 95% confidence intervals for AUC and AP were calculated using 1,000 bootstrap resamples.

The global model performances varied across the different machine learning algorithms evaluated on the training/internal validation cohort (**Figure 2A**). Among all models, ensemble-based approaches generally demonstrated superior discriminatory performance. XGBoost achieved the highest mean AUC of 0.932 (95% CI: 0.920-0.944) and AP of 0.852 (95% CI: 0.826-0.878), followed by AdaBoost, which achieved an AUC of 0.894 (95% CI: 0.881-0.907) and an AP of 0.774 (95% CI: 0.746-0.803). In contrast, instance-based models such as SVM and KNN showed relatively lower performance. Similar trends were observed for AP, reinforcing the advantage of ensemble methods in this task (**Figure 2B**). Pairwise comparisons of AUCs using DeLong’s test showed that the AUC of XGBoost was significantly higher than that of AdaBoost (*P* < 0.001) as well as those of the remaining models (all *P* < 0.0001), supporting the selection of XGBoost as the highest-performing algorithm.

Independent held-out validation further confirmed the generalizability of these models. XGBoost maintained strong performance (AUC = 0.954, (95% CI: 0.937-0.968); AP = 0.903, (95% CI: 0.868-0.933)), while AdaBoost also showed stable results. LR and RF demonstrated moderate discriminative ability, whereas SVM and KNN exhibited notable declines in predictive metrics (**Figures 2C, D**). These results highlight the varying capabilities of different algorithms in distinguishing between DN and DR using structured clinical data. The superior performance of ensemble models, particularly XGBoost, may be attributed to their ability to capture nonlinear patterns and interactions among clinical variables, even in moderately sized datasets. This performance consistency across internal and external cohorts suggests potential clinical utility in stratifying microvascular complications based on routinely collected diagnostic information.

### 3.3 Predictive factors for DN and DR

**Figure 3:**
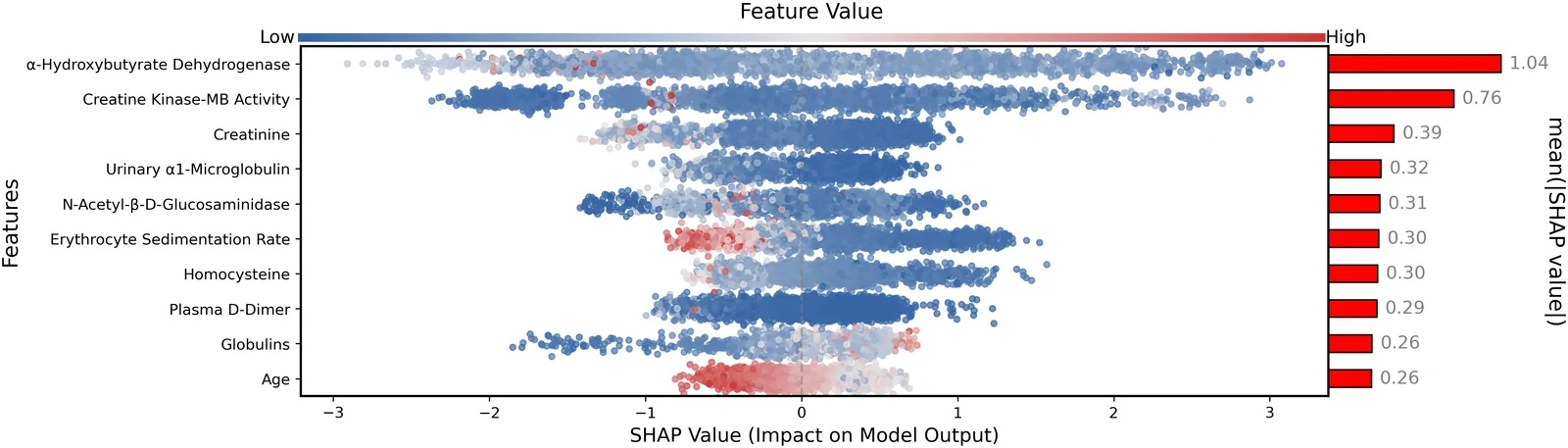
Feature importance ranked by SHAP additive explanations for the top 10 predictors in the XGBoost model. The width of the horizontal bars represents the contribution of each feature to the model’s prediction; a wider bar indicates a greater contribution. The color of the bars reflects the feature magnitude, with a gradient from sapphire blue (low) to brick red (high), as shown in the color bar at the top. The *x*-axis direction indicates the likelihood of developing diabetic nephropathy (DN) (right) or diabetic retinopathy (DR) (left).

To interpret which key predictive factors were most important for differentiating between two diabetic complications, SHAP values were calculated using the top-performing XGBoost model with all 47 clinical parameters. SHAP values quantify the marginal impact of each feature on the model’s predictions, considering the contributions of all other features. To visualize these effects, SHAP plots were generated, with the color gradient denoting the relative values of each feature and the horizontal axis reflecting their directional impact on the predicted risk of different complications. As shown in **Figure 3**, the top ten predictors with the greatest contribution to the classification between DN and DR are highlighted. In detail, α-hydroxybutyrate dehydrogenase (α-HBDH) emerged as the strongest predictor, followed by creatine kinase-MB (CK-MB) and creatinine, all of which are closely related to metabolic disturbances and renal impairment in diabetes. Other notable predictors include urinary α1-microglobulin (Uα1m), an early indicator of tubular injury, N-acetyl-β-D-glucosaminidase (NAG), which is associated with glomerular dysfunction, and erythrocyte sedimentation rate (ESR), a nonspecific marker of systemic inflammation that underscores the inflammatory processes driving diabetic complications. Additionally, homocysteine, reflecting endothelial dysfunction, plasma D-dimer, a marker of thrombotic activity, as well as globulins and age, also contributed significantly, highlighting the multifactorial nature of risk in both DN and DR.

**Figure 4:**
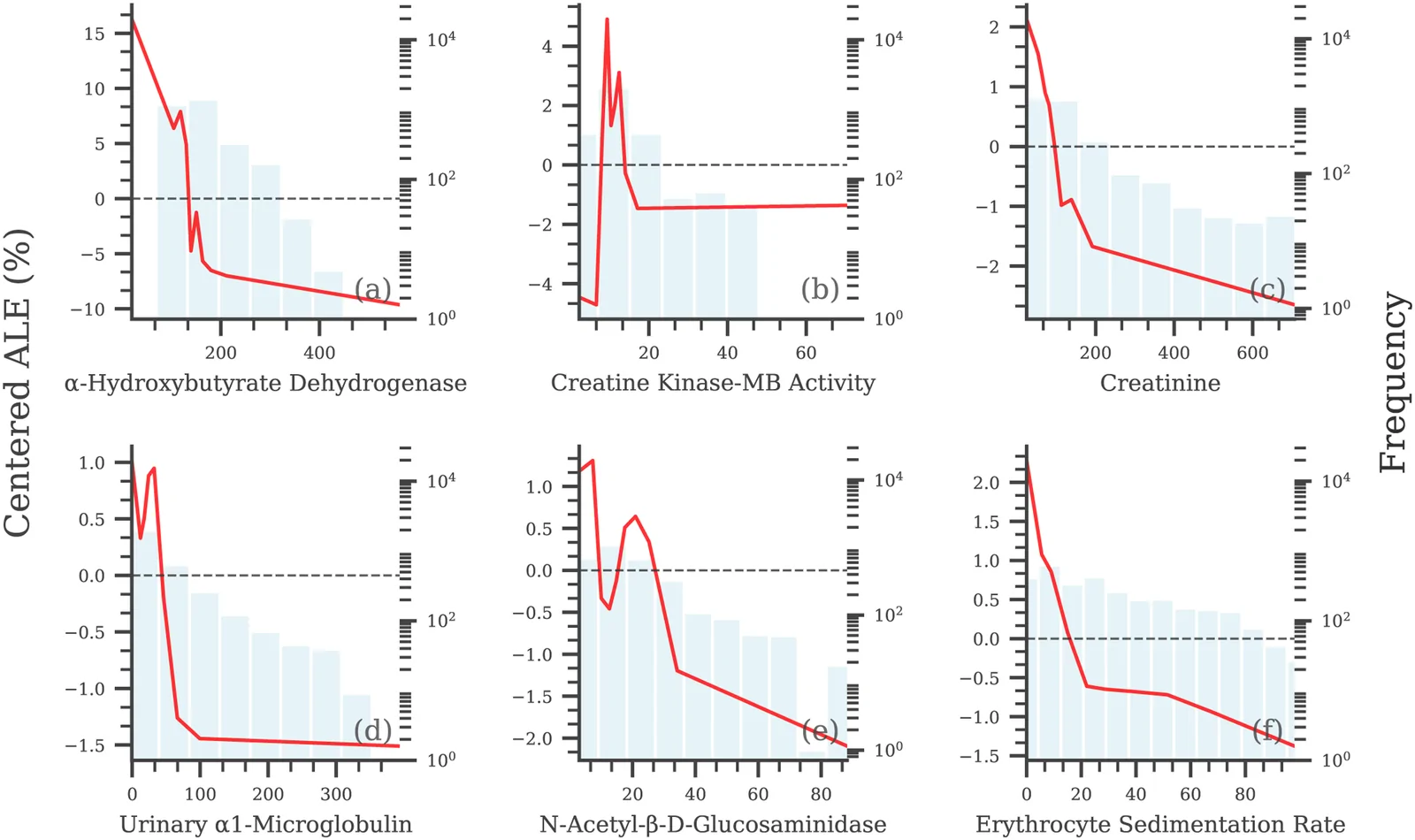
Risk effects and key biomarker profiles in two diabetic complications. ALE plots of the six most important predictors, illustrating how variations in their values shift the predicted risk toward DN or DR. Positive ALE values indicate higher DR risk, while negative values indicate higher DN risk.

Accumulated Local Effects (ALE) analysis was further conducted to examine how the top predictors influenced the likelihood of developing DN and DR. Unlike simple feature ranking, ALE curves enabled the characterization of how gradual changes in each biomarker affected the predicted probability of DN versus DR across its entire value range. In this analysis, ALE values above 0 indicated an increased risk of DR, whereas values below 0 signified an increased risk of DN. α-HBDH exhibited a clear monotonic pattern, with higher levels consistently associated with increased DN risk (**Figure 4**). Similar trends were observed for creatinine, Uα1m, and ESR, underscoring their strong association with renal dysfunction and systemic inflammation. In contrast, CK-MB and NAG showed more complex, non-monotonic effects. For CK-MB, rising levels initially reduced DN risk while elevating DR risk, but beyond a certain threshold, the trend reversed. NAG exhibited an opposite trajectory, where increasing values first increased DN risk and decreased DR risk, before the effect switched within a defined interval. These crossover patterns highlight the competing biological mechanisms underlying DN and DR.

### 3.4 Construction and validation of a final prediction model with clinical benefit assessment for diabetic complications

**Figure 5:**
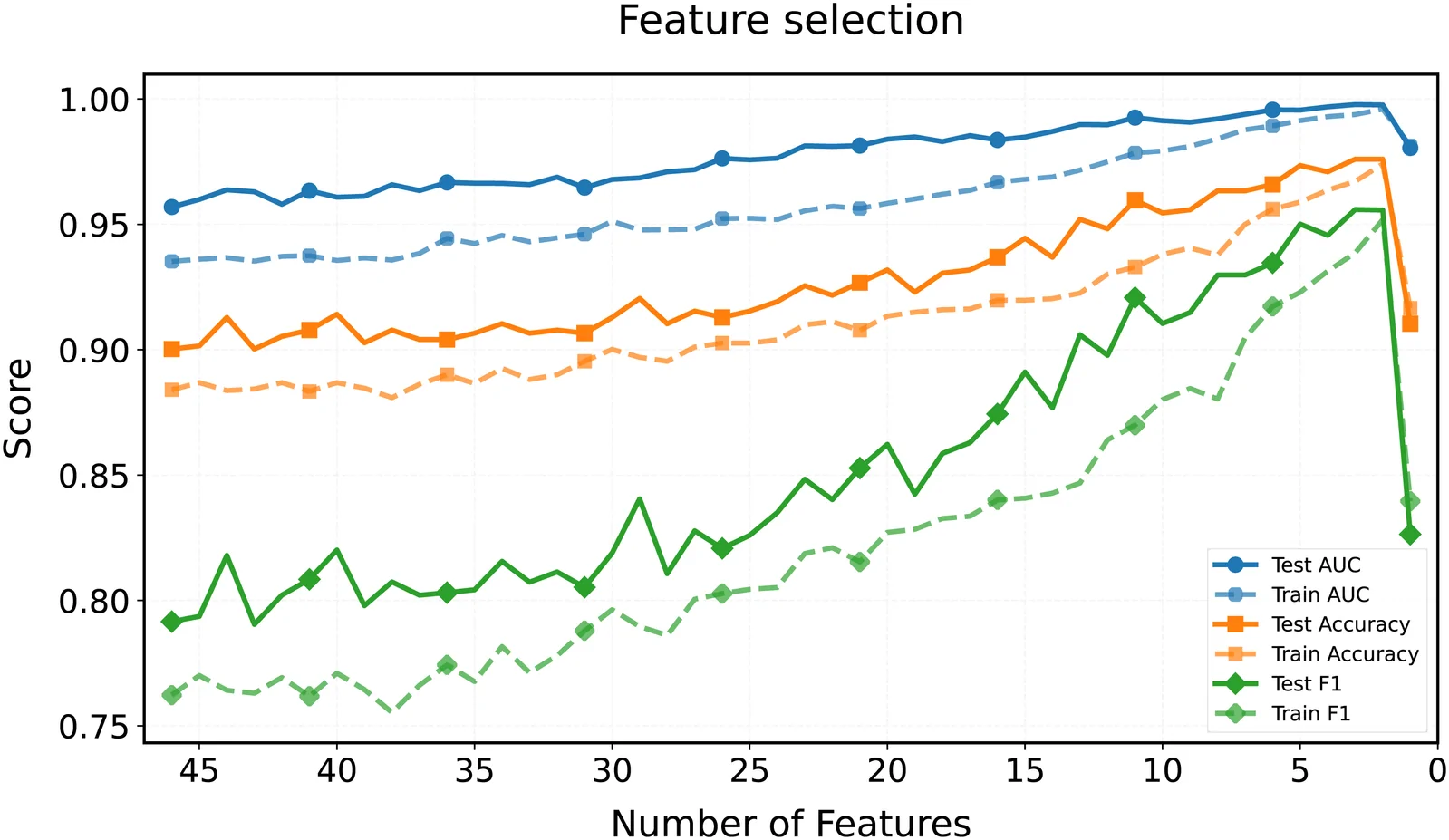
Variable sensitivity analysis with the XGBoost model. The models were established on the training/internal validation cohort. For each model, variables were selected by starting with the maximum number of variables (n=47) and keeping the highest n-1 variables according to their importance in the model. Accuracy, AUC and F1-values were computed at each stage.

To develop accurate and reliable risk prediction models, it is crucial to select the most relevant features that capture key patterns across various diabetic complications. To optimize model performance and enhance clinical applicability, the RFE method was conducted to assess and rank the importance of the initial 47 clinical parameters in the XGBoost model, using the training/internal validation cohort. This approach works by recursively ranking variables based on their importance, eliminating the least significant ones, and continuing this process until the optimal subset of variables is identified. As shown in **Figure 5**, the model’s performance steadily improved as the number of variables decreased, eventually plateauing with five key variables that provided the most stable and efficient results, balancing complexity and predictive power.

**Figure 6:**
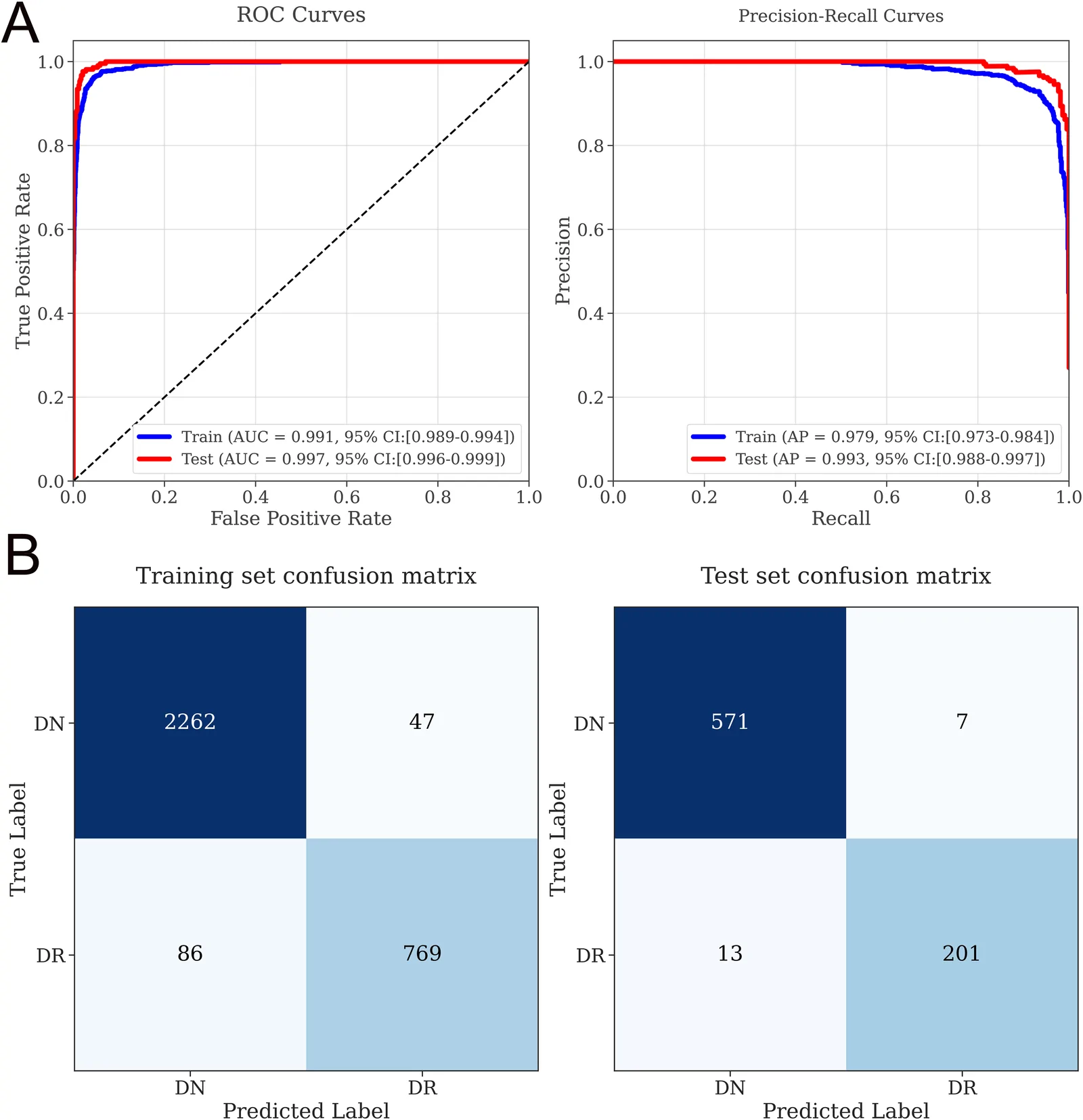
Performance evaluation of the optimized risk prediction model. **(A)** ROC and PR curves demonstrate the discriminative power of the XGBoost model using the selected optimal variables. **(B)** Confusion matrices compare model predictions with ground-truth labels, for both training/internal validation (left) and independent held-out validation (right) cohorts.

Based on the five highest-ranking variables identified through RFE, a final predictive model was developed and evaluated using α-HBDH, CK-MB, creatinine, Uα1m, and NAG. These selected variables were consistent with the most discriminative predictors identified in the SHAP analysis. Together, these findings highlight the robustness and generalizability of the selected predictors. The final model, optimized with parameters including a learning rate of 0.1916, a maximum depth of 3, and 500 estimators, demonstrated high apparent discriminatory performance, achieving an AUC of 0.991 (95% CI: 0.989-0.994) and an AP of 0.979 (95% CI: 0.973-0.984) in the training/internal validation cohort, and an AUC of 0.997 (95% CI: 0.996-0.999) and an AP of 0.993 (95% CI: 0.988-0.997) in the independent held-out validation cohort (**Figure 6A**). To assess whether model performance was primarily driven by renal-related variables, we performed a sensitivity analysis by sequentially excluding such predictors from the original final model. The original model (Model A), including α-HBDH, CK-MB, creatinine, Uα1m, and NAG, achieved an AUC of 0.996 and a Brier score of 0.028. After exclusion of creatinine (Model B), the model achieved an AUC of 0.995 and a Brier score of 0.030. After exclusion of the broader renal/tubular marker panel, leaving only α-HBDH and CK-MB (Model C), the model achieved an AUC of 0.998 and a Brier score of 0.020. Calibration curves for all three models are presented in **Supplementary Figure 1**. These results represent a significant improvement over the model trained on the complete set of variables. Decision curve analysis showed that using the model based on these optimal features to guide clinical decisions provided a favorable net benefit across a clinically relevant range of threshold probabilities (**Supplementary Figure 2**). Specifically, in the training/internal validation cohort (n = 3,164), only 133 incorrect predictions (4.2%) were observed, whereas in the independent held-out validation cohort (n = 792), 20 incorrect predictions (2.5%) were recorded (**Figure 6B**). These relatively low misclassification rates highlight the potential of the optimized model to reduce diagnostic errors and improve decision-making. By establishing these optimized features, the model becomes not only more accurate but also more clinically actionable, providing a practical tool for healthcare professionals to identify patients at risk for DN and DR. Collectively, these findings support the model’s applicability in real-world clinical settings and its potential to facilitate timely interventions.

### 3.5 Personalized risk prediction of DN and DR

**Figure 7:**
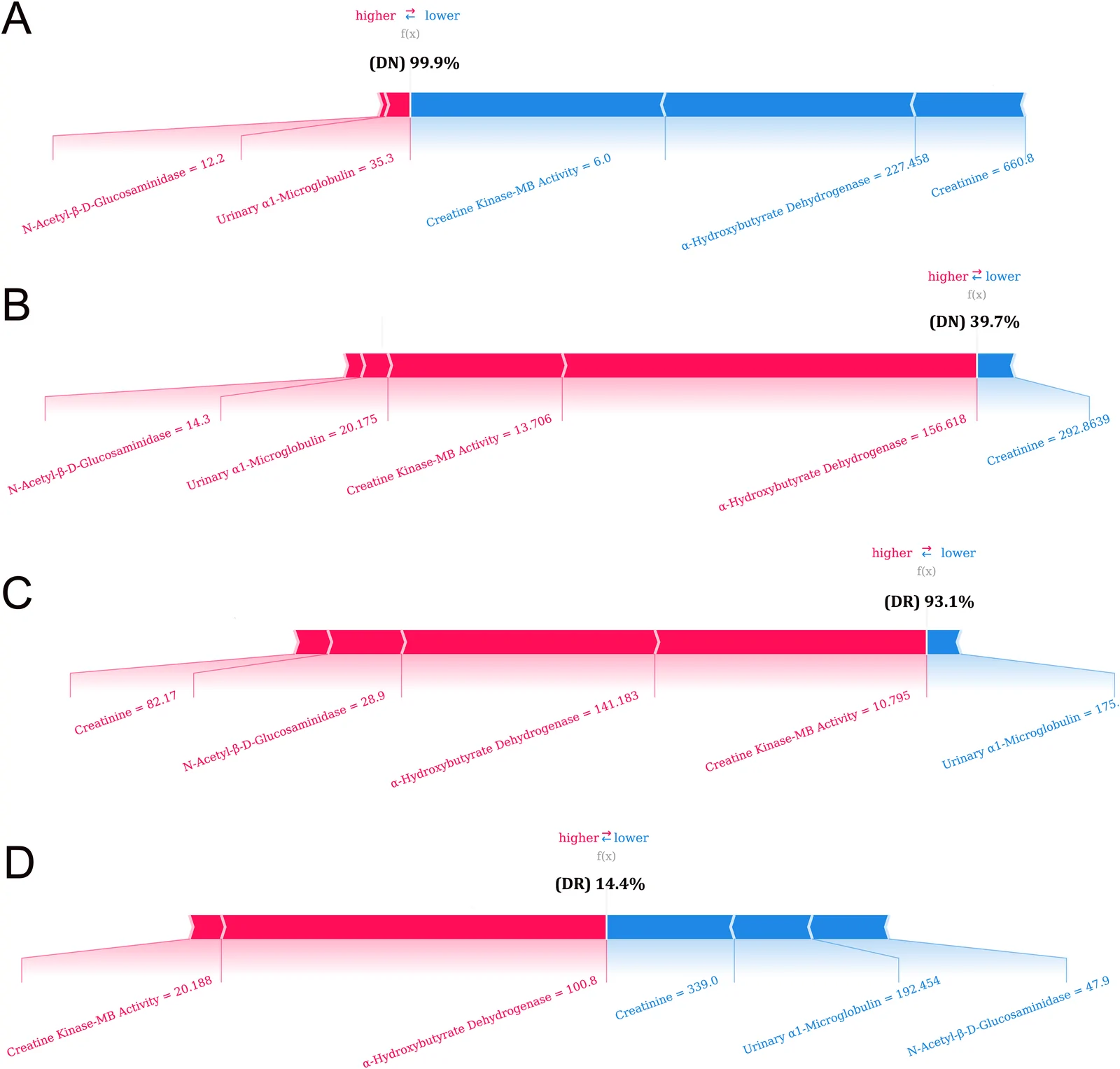
Individual complication profiles interpreted using the optimized risk prediction model. Four representative patients were diagnosed with DN **(A, B)** or DR **(C, D)**, respectively. SHAP force plots illustrate the five most influential predictors contributing to the individualized risk estimates. Red areas denote features driving a higher predicted risk of DR, whereas blue areas denote features driving a higher predicted risk of DN. Numeric values within each area correspond to the observed values of the respective features.

Interpretation of the risk predictive model using SHAP values enabled quantitative assessment of how each patient’s clinical and biomarker data contributed to the predicted risk of developing diabetic complications. To facilitate individualized understanding, patient-specific complication risk profiles were visualized as SHAP force plots, where each biomarker’s positive (risk-increasing) or negative (risk-decreasing) influence was clearly displayed (**Figure 7**). In these plots, features favoring DR and DN were highlighted in red and blue, respectively, providing direct insight into the directionality of each factor. For example, one patient who ultimately developed DN showed an extremely high predicted DN risk (99.9%), primarily driven by markedly elevated levels of α-HBDH and creatinine (**Figure 7A**). In contrast, another patient who also progressed to DN had a much lower predicted DN risk (39.7%). SHAP analysis revealed that, despite a high creatinine level, other biomarkers including α-HBDH, CK-MB, Uα1m, and NAG collectively contributed more strongly toward DR risk (**Figure 7B**). Similar patterns were observed in two additional patients who developed DR, for whom the predicted DR risks were 93.1% and 14.4%, respectively. For the third patient, all predictors except Uα1m supported DR risk, which explained the high predicted probability (**Figure 7C**). Conversely, for the fourth patient, elevated creatinine and Uα1m levels strongly favored DN, leading to a substantially reduced predicted DR probability of 14.4%, despite the patient’s eventual DR diagnosis (**Figure 7D**). Taken together, these findings demonstrate that SHAP-based interpretability not only provides individualized explanations for predictions but also clarifies how distinct biomarker combinations influence predicted outcomes. Such personalized profiles may facilitate the identification of heterogeneous disease patterns and improve risk stratification strategies for diabetic complications.

## 4 Discussion

In this study, a complication classification model was developed for hospitalized patients with T2DM using seven machine learning algorithms based on routine clinical test variables. The models demonstrated variable diagnostic performance, with AUC values ranging from 0.617 to 0.954. Among them, the XGBoost model achieved the highest overall accuracy (AUC: 0.954, 95% CI: 0.937-0.968) and superior precision-recall performance (AP: 0.903, 95% CI: 0.868-0.933). Feature selection further enhanced the model’s efficiency and stability by addressing issues associated with high-dimensional input, such as prolonged training time and overfitting. SHAP analysis identified the top discriminative features driving complication subtype classification. These findings support the potential of interpretable machine learning models to assist in timely and accurate stratification of complication types in hospitalized patients with T2DM.

DN and DR are among the most prevalent microvascular complications of T2DM and represent key targets for early risk stratification and individualized clinical management. However, few studies have investigated the differentiation of these two complications using routinely collected clinical laboratory data. Most existing AI models have focused primarily on predicting the onset of T2DM, with reported diagnostic accuracies ranging from 0.80 to 0.99 (35, 36). For instance, Li et al. developed a machine learning framework based on blood routine datasets, achieving excellent performance, while also employing SHAP to identify important predictive features, highlighting the potential of AI in aiding timely and interpretable diabetes diagnoses (37). In contrast, the present study developed a predictive model for complication type based on routine inpatient data and achieved strong apparent discriminative performance. The final XGBoost model yielded an AUC of 0.991 in the training/internal validation cohort and 0.997 in the independent held-out validation cohort, comparable to or exceeding previously published results. This high level of accuracy and generalizability may be attributed to the algorithm’s ensemble structure, which enhances model robustness through sequential boosting and iterative error minimization.

Traditional methods for the early diagnosis and severity assessment of DN and DR typically rely on renal biopsy or fundus imaging, both of which are limited by invasiveness, operator dependence, or low sensitivity in early stages (25–27). These approaches are subject to interobserver variability and inconsistent interpretation, underscoring the need for objective, reproducible diagnostic tools that minimize reliance on subjective judgment. To address this challenge, the present study developed the final model that classified complication subtypes in T2DM using routinely collected clinical laboratory data. With the growing availability of EHRs and biomarker-based indicators, ML has shown strong potential in extracting clinically relevant patterns from complex datasets (38–40). However, excessive feature input may introduce noise and overfitting, compromising model performance and generalizability. In this study, RFE was applied to identify the most informative variables, resulting in a simplified XGBoost model with five key features. By maintaining high diagnostic accuracy with a reduced feature set, this approach improves model efficiency and robustness while reducing clinical complexity. These advantages may translate into more targeted laboratory testing and more consistent diagnostic decision-making, ultimately supporting cost-effective, high-quality care for patients with T2DM-related complications.

As the application of machine learning in medicine continues to expand, offering a powerful means to identify individuals at risk for complications, concerns regarding model interpretability have become increasingly prominent. Many frontline clinicians encounter difficulties in understanding the complex internal logic of these algorithms, resulting in limited trust in their outputs-a challenge commonly referred to as the “black box” problem (20). To enhance interpretability in clinical contexts, SHAP has emerged as a widely adopted tool for quantifying the contribution of individual features to model predictions. In this study, SHAP was applied to interpret the decision-making process of the final XGBoost model, identifying α-HBDH, CK-MB, creatinine, Uα1m, and NAG as the five most influential features contributing to complication subtype classification. Comparative SHAP visualizations between DN and DR cases further revealed distinct feature contribution patterns, providing clinicians with greater transparency and highlighting key laboratory parameters relevant to early complication differentiation. The biological plausibility of the selected features is supported by their relationship to known manifestations of diabetic complications. Creatinine reflects impaired glomerular filtration, whereas Uα1m and NAG are markers of tubular dysfunction and injury, consistent with the renal involvement characteristic of DN. The mean absolute SHAP values ranked α-HBDH (1.04) and CK-MB (0.76) substantially higher than the remaining variables, with α-HBDH contributing approximately 2.7-fold more than creatinine (0.39) and CK-MB about 2-fold more; the other predictors were clustered within a narrower range (0.26-0.32). Because α-HBDH and CK-MB are not renal-specific biomarkers and are partly influenced by renal clearance, their strong contributions to the discrimination between DN and DR may partly capture differences in renal functional status. Accordingly, the identification of DR cases by the model may reflect not only retinopathy-related clinical characteristics, but also the relative absence of the renal injury-related biochemical profile more typical of DN. This interpretation is biologically plausible, as both DR and DN are microvascular complications of diabetes, while renal laboratory abnormalities are expected to be more pronounced in DN.

Although the development of a complication prediction model based solely on routine clinical laboratory data yielded promising results, several limitations should be acknowledged. First, the training and validation cohorts were derived from the same institution, and therefore the results may reflect institution-specific patient characteristics, laboratory measurement procedures, and clinical workflows. Potential overfitting or limited transportability cannot be fully excluded. Future studies using independent multicenter and prospective cohorts are required to confirm the robustness, calibration, and clinical applicability of the model. Second, the model focused exclusively on DN and DR, excluding other clinically relevant complications such as diabetic foot ulcers, cardiovascular disease, and diabetic neuropathy, which may share overlapping risk factors and pathophysiological mechanisms. An additional important limitation is that the present model was developed as a binary classifier to distinguish DN from DR. However, in real-world clinical practice, diabetic microvascular complications may coexist, and many patients can present with both nephropathy and retinopathy. Therefore, the current model should not be interpreted as excluding the possibility of overlapping complications. For patients with clinical suspicion of both DN and DR, standard parallel diagnostic pathways, including renal assessment and ophthalmic examination, remain necessary. As shown in **Table 1**, several baseline clinical and laboratory variables differed between the DN and DR groups, indicating differences in case-mix. In addition, some predictors in the original final model, particularly renal function and tubular injury markers, may partially overlap with the clinical evaluation used to distinguish DN from DR, raising concern about outcome-definition leakage. However, sensitivity analyses showed that model performance was not reduced after excluding creatinine and further improved after excluding the broader renal/tubular marker panel, suggesting that the model’s discriminative ability was not dependent on renal-related markers. Third, several key clinical indicators were not included, such as imaging-derived parameters, omics-based biomarkers, and longitudinal data (e.g., HbA1c variability, disease duration), all of which could further enhance predictive accuracy and clinical applicability. An important clarification is that the current model was developed among patients who had already been diagnosed with either DN or DR. Therefore, it addresses a differential-classification task rather than an early-detection or onset-prediction task. Because no complication-free comparator group was included, the model cannot be used to estimate the risk of developing DN or DR, detect asymptomatic complications, or screen the general T2DM population. Future studies including complication-free patients with longitudinal follow-up are required if incident complication prediction is intended. Lastly, complication labels were derived from existing diagnostic records rather than standardized gold-standard assessments, and variables with substantial missingness were excluded, potentially omitting informative predictors. Future research shall focus on multi-center validation, multimodal imaging-omics integration, and prospective real-world evaluation of model utility, interpretability and cost-effectiveness. Multi-label/multi-task models for concurrent prediction of DN, DR and their co-occurrence should be developed to reflect the complexity of diabetic complications.

In conclusion, the interpretable ML model developed using routine clinical laboratory data represents a practical and scalable diagnostic tool for differentiating complication subtypes in hospitalized patients with T2DM. By identifying individuals who require tailored management strategies, the model supports more precise clinical decision-making, reduces diagnostic delays, and facilitates earlier intervention. Designed for seamless integration with hospital informatics systems, it holds promise for enhancing care delivery in community and resource-limited settings, where access to specialist diagnostics may be constrained. Nonetheless, further prospective validation across diverse clinical environments is warranted to confirm its generalizability and real-world impact.

## Data Availability

The original contributions presented in the study are included in the article/**Supplementary Material**. Further inquiries can be directed to the corresponding authors.
